# A single amino acid substitution in aromatic hydroxylase (HpaB) of *Escherichia coli* alters substrate specificity of the structural isomers of hydroxyphenylacetate

**DOI:** 10.1186/s12866-020-01798-4

**Published:** 2020-05-06

**Authors:** Hanseol Kim, Sinyeon Kim, Dohyeon Kim, Sung Ho Yoon

**Affiliations:** Department of Bioscience and Biotechnology, Konkuk University, Seoul, 05029 Republic of Korea

**Keywords:** Hydroxyphenylacetic acid, 4-hydroxyphenylacetate 3-hydroxylase, Structural isomer, Substrate specificity

## Abstract

**Background:**

A broad range of aromatic compounds can be degraded by enteric bacteria, and hydroxyphenylacetic acid (HPA) degrading bacteria are the most widespread. Majority of *Escherichia coli* strains can use both the structural isomers of HPA, 3HPA and 4HPA, as the sole carbon source, which are catabolized by the same pathway whose associated enzymes are encoded by *hpa* gene cluster. Previously, we observed that *E. coli* B REL606 grew only on 4HPA, while *E. coli* B BL21(DE3) grew on 3HPA as well as 4HPA.

**Results:**

In this study, we report that a single amino acid in 4-hydroxyphenylacetate 3-hydroxylase (HpaB) of *E. coli* determines the substrate specificity of HPA isomers. Alignment of protein sequences encoded in *hpa* gene clusters of BL21(DE3) and REL606 showed that there was a difference of only one amino acid (position 379 in HpaB) between the two, viz., Arg in BL21(DE3) and Cys in REL606. REL606 cells expressing HpaB having Arg379 could grow on 3HPA, whereas those expressing HpaB with Gly379 or Ser379 could not. Structural analysis suggested that the amino acid residue at position 379 of HpaB is located not in the active site, but in the vicinity of the 4HPA binding site, and that it plays an important role in mediating the entrance and stable binding of substrates to the active site.

**Conclusions:**

The arginine residue at position 379 of HpaB is critical for 3HPA recognition. Information regarding the effect of amino acid residues on the substrate specificity of structural isomers can facilitate in designing hydoxylases with high catalytic efficiency and versatility.

## Background

Hydroxyphenylacetic acid (HPA) is an aromatic compound that is abundantly present in nature [1, 2]. Its structural isomers, 3HPA and 4HPA, are phenylacetic acids in which a hydroxy group substitutes the hydrogen atom at the meta and para positions on the benzene ring (Additional file 1: Figure S1). In the human intestine, 3HPA and 4HPA are the major metabolites produced during the degradation of flavonoids, proanthocyanidin [3] and kaempferol [4], respectively. Further, 4HPA is the main product of L-tyrosine fermentation in the intestine [2]. Additionally, 4HPA has been proposed as a candidate hepatoprotective drug [5] and a biological marker for depression and anxiety [6]. Furthermore, 4HPA can be derived from the biodegradation of lignin, which is an abundant component of the lignocellulosic biomass [7, 8].

Aromatic compounds are predominantly degraded by bacteria and fungi [1]. Most enteric bacteria can use HPA as a carbon source, with *E. coli* being the most studied [2, 8]. Among the laboratory strains of *E. coli*, *E. coli* B, C, and W can grow on 3HPA and 4HPA, whereas *E. coli* K-12 cannot [9]. In *E. coli*, both compounds are catabolized via the homoprotocatechuate (3,4-hydroxyphenylacetate) (HPC) pathway and are subsequently converted into pyruvate and succinate. The *hpa* gene cluster contains 8 genes which are organized into *hpaBC* (HPA hydroxylase operon) and *hpaGEDFHI* (HPC *meta*-cleavage operon), two regulatory genes (*hpaR* and *hpaA*), and *hpaX* encoding the HPA transporter [10] (Additional file 1: Figure S2). As the G + C content of the *hpa* cluster and the *E. coli* genome is similar, and as most enteric bacteria can utilize 3HPA and 4HPA, the lack of growth of *E. coli* K-12 on HPA might be due to the loss of the *hpa* cluster present in the ancestors (Additional file 1: Figure S2) [2].

Both 3HPA and 4HPA are hydroxylated to HPC by the 4-hydroxyphenylacetate 3-monooxygenase complex (HpaBC), which catalyzes the initial step in aerobic HPA catabolism [2]. The complex is a flavin adenine dinucleotide (FAD)-dependent hydroxylase consisting of a monooxygenase (HpaB) and flavin reductase (HpaC) [11]. Although HpaB requires the reduced FAD supplied by HpaC, when only HpaB was expressed without any concurrent expression of HpaC in *E. coli* K-12, it was able to show hydroxylating activity [12]. *E. coli* HpaB can hydroxylate a broad range of phenolic compounds, from simple phenol to complex phenylpropanoids [2, 13, 14]. The crystal structure of *E. coli* HpaB has been recently determined, suggesting that a unique loop structure covering the active site is essential for the catalytic versatility [15].

The *E. coli* B lineages, BL21(DE3) and REL606 have long been used for numerous biotechnological applications and long-term experimental evolution, respectively [16]. In our previous studies, the two strains showed different utility of HPA as the sole carbon source. BL21(DE3) could grow on both isomers, 3HPA and 4HPA [17]; however, REL606 could grow only on 4HPA [18]. In this study, we report that a single amino acid residue in HpaB is responsible for the altered substrate specificity of the HPA isomers. The single nucleotide of the corresponding amino acid was subjected to site-directed mutagenesis to provide experimental evidences. Based on protein structure homology modeling and substrate docking simulation, the single amino acid residue was shown to have structural importance in recognizing 3HPA but not 4HPA.

## Results

### Identification of a single amino acid change in HpaB of *E. coli* REL606

**Fig. 1:**
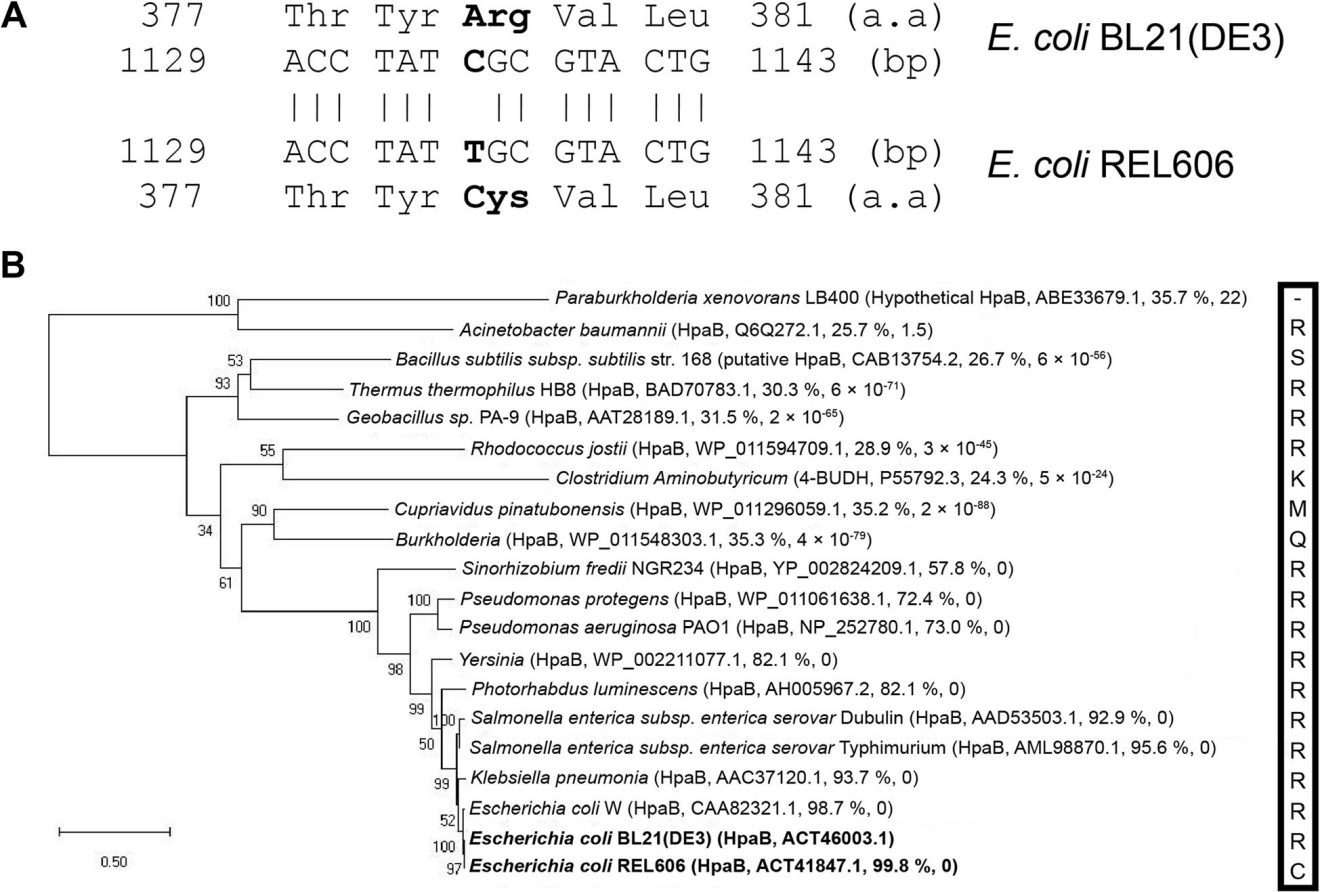
Sequence alignment of HpaB homologs. **a** Pairwise sequence alignment between HpaBs from *E. coli* BL21(DE3) and REL606 at the nucleotide and protein sequence levels. The aligned nucleotides were identical except for those at position 1135 of *hpaB*. **b** Phylogenetic tree of HpaB homologs. Multiple alignments of 20 protein sequences were performed using MUSCLE [19]. The maximum likelihood tree was built using MEGA X with bootstraps of 1000 replicates [20]. Bootstrap values (as percentages) are denoted at internal nodes. For each HpaB homolog of *E. coli* BL21(DE3), the BLASTP result is given as the accession number, amino acid identity, and e-value. Scale bar indicates substitution per amino acid sequence site. Amino acid residues at position 379 are shown in the box at the right

Our previous phenotype microarray tests of the two *E. coli* B strains had revealed that REL606 utilized only 4HPA [18], whereas BL21(DE3) utilized both 3HPA and 4HPA [17]. We first checked whether there is any difference in sequence between *hpa* gene clusters of these strains. Pairwise sequence alignments of 11,127-bp long genomic regions encompassing 11 *hpa* genes revealed that only two nucleotides were different in protein coding sequences between the two strains. Compared to BL21(DE3), REL606 exhibited one non-synonymous substitution in *hpaB* (C → T at 1135th bp downstream of the start codon and arginine→cysteine at 379th amino acid residue) (Fig. 1a) and one synonymous substitution in *hpaH* (C → T at 195th bp downstream of the start codon).

Intrigued by the amino acid difference in HpaB, we performed phylogenetic analysis of 20 HpaB homologs (Fig. 1b). The reconstructed phylogenetic tree was in agreement with a previous report [11]. In most cases (14 out of 20), arginine occupied the position 379 of HpaB. Interestinlgy, this arginine residue was conserved in all enteric bacteria but not in REL606.

The historical origin of the REL606 and BL21(DE3) has been well-documented [21]. The two *E. coli* B strains had a common ancestor sometime between 1942 and 1959 and went through different sets of genetic manipulations [22]. Detailed comparison of the genomic sequences of BL21(DE3) and REL606 provided plausible explanation for every single base-pair difference [22]. Evidently, the C-to-T transition in *hpaB* was caused by 1-methyl-3-nitro-1-nitrosoguanidine (MNNG)-mediated mutagenesis of the REL606 progenitor, and subsequently, the mutation became unintentionally fixed through single-colony isolation.

### Growth of REL606 on 3HPA conferred by expression of BL21(DE3)-derived *hpaB*

The simplest explanation for the inability of *E. coli* REL606 to catabolize 3HPA could be the replacement of Arg379 (in HpaB of BL21(DE3)) with Cys379. To test this hypothesis, we constructed vectors constitutively expressing the *hpaB* of BL21(DE3) [pHCE-IIB-HpaB(R379)] and *hpaB* of REL606 [pHCE-IIB-HpaB(C379)], and transformed them into REL606.

**Fig. 2:**
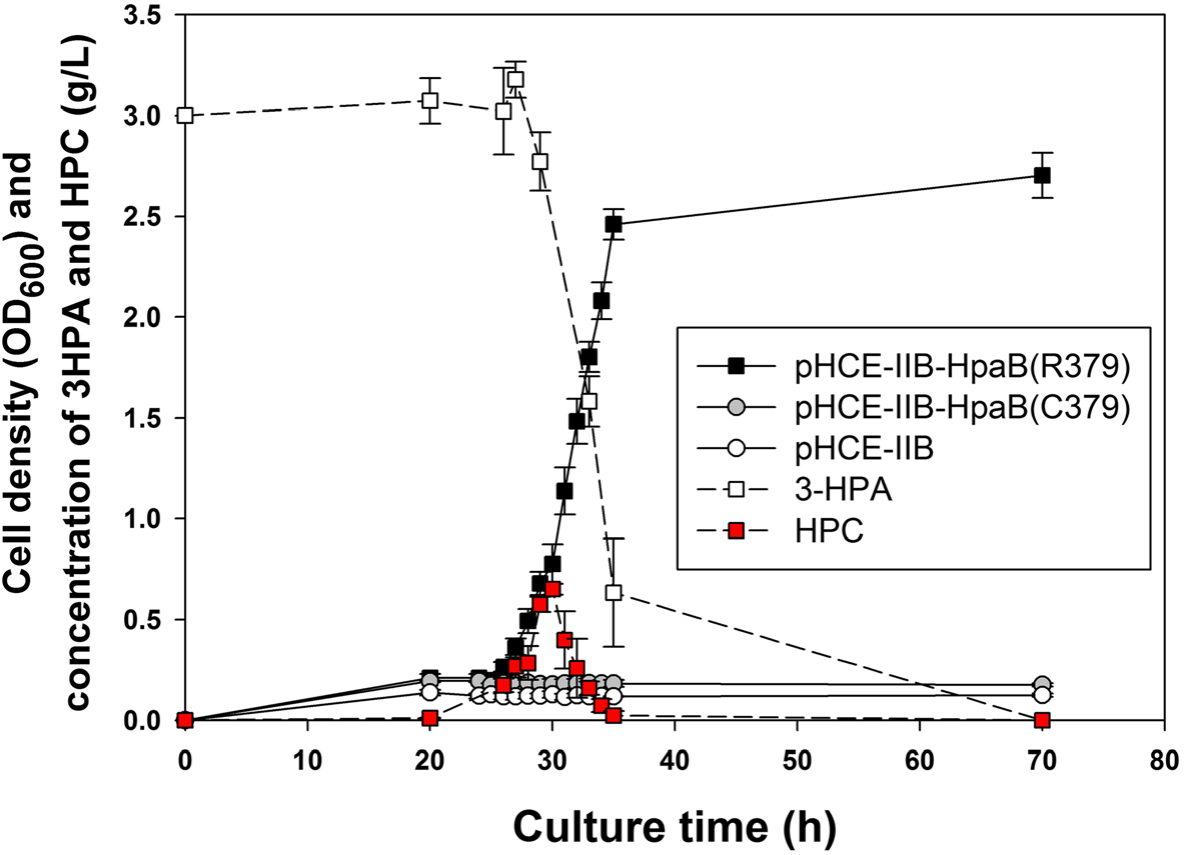
Growth curves of *hpaB*-expressing REL606 in defined medium with 3HPA as sole carbon source. *E. coli* REL606 cells harboring expression constructs BL21(DE3)-derived *hpaB* [pHCE-IIB-HpaB(R379), ] and REL606-derived *hpaB* [pHCE-IIB-HpaB(C379), ] are shown. REL606 harboring the control plasmid (pHCE-IIB, ) was used as the control. The culture medium comprised defined medium supplemented with 3 g/L (20 mM) 3HPA. Only REL606 harboring pHCE-IIB-HpaB(R379) grew on 3HPA, and concentrations of 3HPA in the culture supernatant (denoted as white squares with a dashed line) and HPC (red squares with a dashed line) were quantified. Error bars denote the standard deviation of mean from three independent cultures

We investigated the effect of BL21(DE3)-derived *hpaB* expression in REL606 on bacterial growth by employing 3HPA as the sole carbon source. Among REL606 harboring each of the three plasmids [pHCE-IIB, pHCE-IIB-HpaB(R379), and pHCE-IIB-HpaB(C379)], only that with pHCE-IIB-HpaB(R379) exhibited growth on defined medium supplemented with 3 g/L of 3HPA (Fig. 2). In the culture supernatant of this strain, the 3HPA concentration decreased with increase in cell density, and extracellular hydroxylated 3HPA (HPC) accumulated up to a maximum concentration of 0.7 g/L after 30 h of incubation. This result demonstrates that inability of REL606 to grow on 3HPA can be completely rescued by the expression of BL21(DE3)-derived *hpaB*.

### Site-directed mutagenesis of HpaB

As substitution of arginine with cysteine in HpaB resulted in *E. coli* that was unable to metabolize 3HPA, we performed site-directed mutagenesis to explore whether replacement with other amino acids has the same effect on substrate specificity. The arginine residue at position 379 of the BL21(DE3)-derived HpaB was replaced with glycine and serine. Glycine is the smallest residue, and the lack of a side group makes glycine the most flexible amino acid, and thus, glycine residue is often located in enzyme active site regions [23]. Serine differs from cysteine only with respect to the switch of sulfur atom with an oxygen and can form a disulfide bond.

**Fig. 3:**
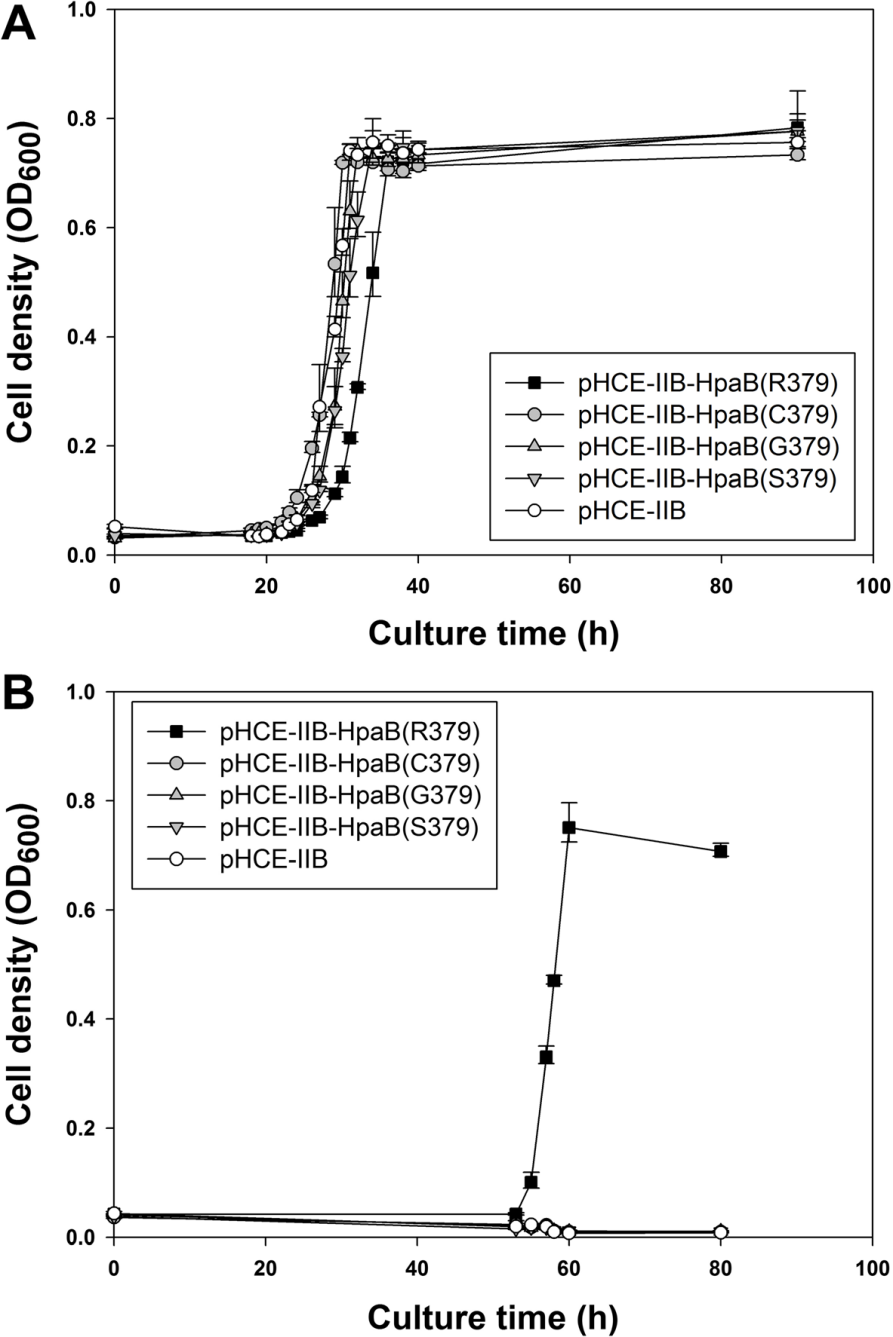
Growth curves of REL606 expressing *hpaB* variants in defined medium with 4HPA (**a**) or 3HPA (**b**) as sole carbon source. *E. coli* REL606 cells harboring expression constructs BL21(DE3)-derived *hpaB* [pHCE-IIB-HpaB(R379), ]; REL606-derived *hpaB* [pHCE-IIB-HpaB(C379), ]; mutant *hpaB*s [(pHCE-IIB-HpaB(G379), ]; and pHCE-IIB-HpaB(S379), ]. REL606 harboring the control plasmid (pHCE-IIB, ) was used as control. The culture medium comprised defined medium supplemented with 0.76 g/L (5 mM) of 3HPA or 4HPA. Error bars denote standard deviation of the mean from three independent cultures

We constructed pHCE-IIB-HpaB(G379) and pHCE-IIB-HpaB(S379) constitutively expressing HpaB with a single amino acid substitution at position 379 (glycine and serine, respectively). Each of the constructed vectors was transformed into *E. coli* REL606. The expression of the HpaB proteins was confirmed by running the proteins on a sodium dodecyl sulfate-polyacrylamide gel electrophoresis (SDS-PAGE) gel (Additional file 1: Figure S3). The gel image showed that endogenous HpaB was hardly detected from REL606 transformed with the empty plasmid and a large amount of the HpaB variants cloned in pHCE-IIB was expressed with culture time. The transformed strains were grown on defined medium supplemented with 0.76 g/L (5 mM) 3HPA or 4HPA as the sole carbon source (Fig. 3). As a comparison, REL606 cells expressing BL21(DE3)-derived *hpaB* [pHCE-IIB-HpaB(R379)] and REL606-derived *hpaB* [pHCE-IIB-HpaB(C379)] were tested. All the strains expressing HpaB with an amino acid substitution at position 379 (Arg, Cys, Gly, or Ser) grew on 4HPA (Fig. 3a). However, when 3HPA was used as the carbon source, only cells expressing HpaB with Arg379 exhibited growth (Fig. 3b). These results suggest that Arg379 in HpaB plays an important role in recognizing 3HPA but not 4HPA.

The strains expressing HpaB with an amino acid substitution at position 379 (Arg, Cys, Gly, or Ser) were also tested for their ability to degrade L-tyrosine (Additional file 1: Figure S4). In the defined medium supplemented with 3 g/L glucose and 0.54 g/L (3 mM) L-tyrosine, brown coloration in the medium was only observed for REL606 expressing the BL21(DE3)-derived *hpaB* gene from pHCE-IIB-HpaB(R379). As catechol derivatives form spontaneous black or brown oxidation products [13], the brown colour of the culture medium is a read-out of HpaB-mediated hydroxylation of L-tyrosine. This result demonstrated that the inability of REL606 to degrade L-tyrosine can be rescued by the expression of BL21(DE3)-derived *hpaB*. Collectively, these results suggest that the arginine residue at position 379 of HpaB is critical for recognition of 3HPA and L-tyrosine.

### Structural importance of the 379th postion in HpaB

**Fig. 4:**
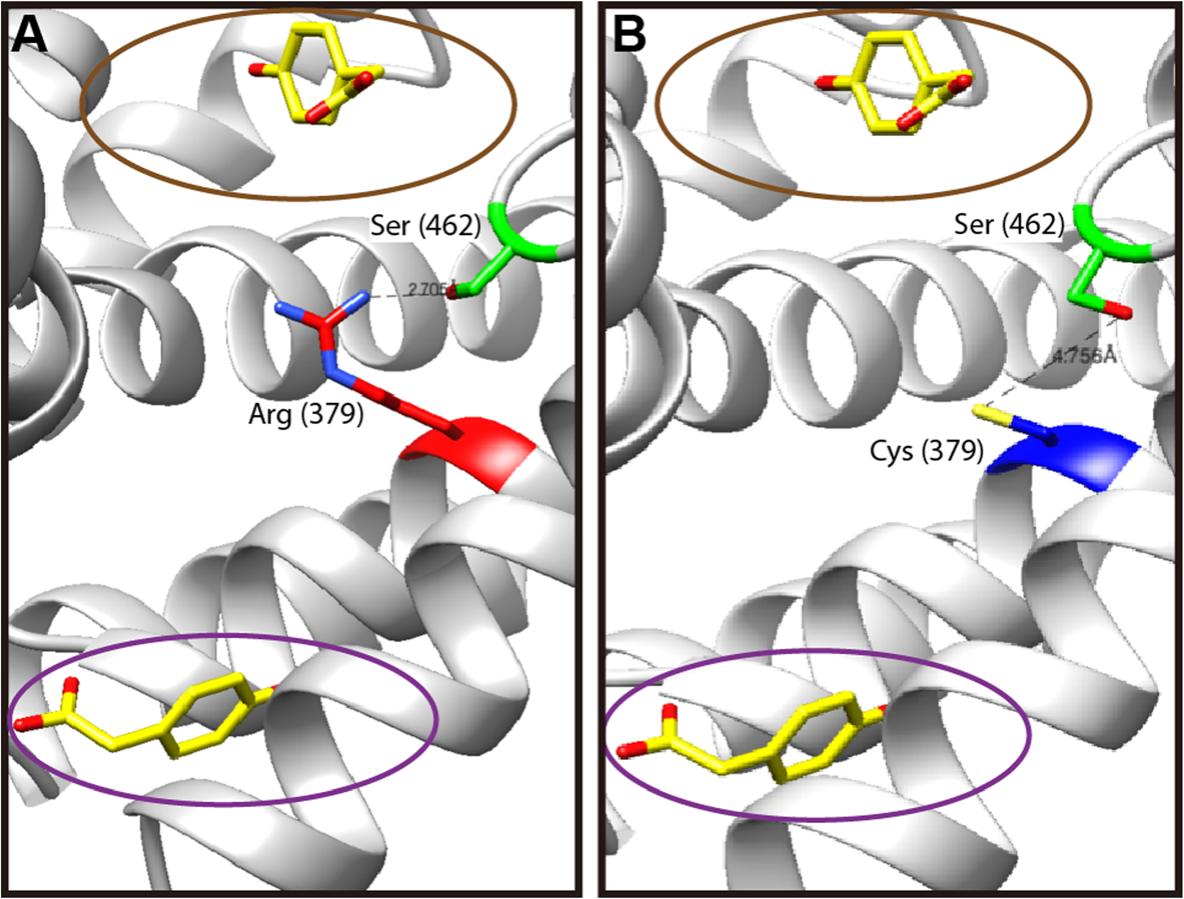
Geometric comparsion of 379th position of *E. coli* HpaB with different amino acid residues. **a** The geometry of Arg379 determined from the published crystal structure of *E. coli* HpaB (PDB ID: 6 EB0). **b** Geometry of Cys379 predicted from homology modeling. The brown circle indicates the site where 4HPA and FAD bind, and the purple circle denotes the extra binding region of 4HPA [15, 24]. 4HPA (colored yellow) bindings were identified using the crystal structure of the HpaB–FAD–4HPA complex from *T. thermophilus* HB8 (PDB ID: 2YYJ). Distance between Ser462 in the C-terminal helical arm to Arg379 or Cys379 were predicted to be 2.70 Å and 4.75 Å, respectively.

So far, the crystal structures of FAD-dependent HpaB were determined as the apoenzyme form from *E. coli* (PDB ID: 6 EB0) [15] and enzyme complex with FAD and 4HPA from *Thermus thermophilus* HB8 (PDB ID: 2YYJ) [24]. To gain structural insights into the importance of the 379th amino acid residue with respect to the recognition of HPAs by HpaB, its structural position was identified using the crystal structure of *E. coli* HpaB apoenzyme form [15], which has the same amino acid sequences as that of HpaB of BL21(DE3) (Fig. 4a). Geometry of Cys379 was predicted by homology modeling based on the crystal structure of HpaB of *E. coli* [15] (Fig. 4b). The active site at which 4HPA binds to was identified using the crystal structure of the HpaB–FAD–4HPA complex from *T. thermophilus* [15, 24].

From the crystal structure of the HpaB-FAD-4HPA complex of *T. thermophilus*, 4HPA was predicted to bind to the active site and an extra binding site in HpaB [24]. Their structural positions were identified using the crystal structure of *E. coli* HpaB apoenzyme form [15]. As shown in Fig. 4, the 379th position was located between the predicted active site [the N-terminal end of α8 (including Arg113, Asp116, and Try117) and the first three amino acids (Asn154, His155, and Ala156) of β3 strand] and the predicted extra binding site [the N-terminal end of α22 (including His372, Ala373, and Ala374) and C-terminal end of α27 (including Ala476, Gln477, and Asn478)]. Interestingly, replacement of Arg379 by Cys379 increased the distance from Ser462 in the C-terminal α-helical arm (Leu456 to Leu519) from 2.70 Å to 4.75 Å, implying a reduced interaction between them. Reportedly, the C-terminal tail of HpaB participates in the formation of the HpaB dimer [15] and a groove which acts as the binding of FADH_2_ and the substrate [24]. The location of the position 379 in the vicinity of the predicted extra binding sites and its reduced interaction with C-terminal tail due to the presence of Cys379 might suggest that Arg379 is optimized for the entrance and stable binding of substrates into the active site. Detailed structural analysis is required to understand how the identified region affects substrate specificity.

The phenol hydroxyl group of 4HPA forms a hydrogen bond with the binding site of HpaB, which is structurally conserved between HpaBs of *E. coli* and *T. thermophilus* [11]. Thus, we reasoned that orientation of the hydroxyl group of HPA may be important in the recognition of the substrate. To predict the conformation and affinity of 3HPA and 4HPA binding to HpaB, the substrates were docked into the active site of the HpaB–FAD–4HPA complex from *T. thermophilus* [24] (Additional file 1: Figure S5). The binding conformation of 4HPA based on the docking simulation matched well with that from the crystal structure [root mean square deviation (RMSD) of the average distance between the backbone atoms of 4HPA was 1.053 Å and the predicted binding affinity was − 6.7 kcal/mol], which validated our docking protocol. However, the RMSD for the simulated docking of 3HPA compared to that of 4HPA from the crystal structure increased to 1.921 Å and the predicted binding affinity decreased to − 6.1 kcal/mol. This suggests that orientations of 3HPA and 4HPA are different, further implying that they may interact with different residues of HpaB when they bind to the active site of HpaB.

## Discussion

In this study, we experimentally demonstrated that a single amino acid substitution in HpaB resulted in the inablility to utilize 3HPA, but did not affect 4HPA utilization. Previously, we found that *E. coli* B REL606 could not grow by utilizing 3HPA as the sole carbon source [18], whereas its closely related B strain, BL21(DE3) could grow on 3HPA as well as on 4HPA [17]. Alignment of protein sequences encoded in the complete *hpa* gene clusters of two *E. coli* B strains revealed that only one amino acid in HpaB was different between the two strains, resulting in a single amino acid change from arginine in BL21(DE3) to cysteine in REL606 at position 379 (Fig. 1a). Constitutive expression of HpaB containing Arg379 resulted in REL606 that was capable of growing on 3HPA (Fig. 2). However, the expression of HpaB containing either Gly379 or Ser379 had no effect on the growth of REL606 on 3HPA or 4HPA (Fig. 3a). Structural modeling of *E. coli* HpaB showed that the 379th position is located not in the active site, but in the vicinity of 4HPA binding sites (Fig. 4), suggesting the important role of Arg379 in mediating the entrance and stable binding of HPA by HpaB. Taken together, these results provide conclusive evidence that the amino acid at position 379 in HpaB of *E. coli* determines the substrate specificity for 3HPA and 4HPA isomers. It is worth noting that this study showcases how genomic and phenomic comparison between closely related strains [17, 18] can lead to unexpected biological discovery at the molecular level.

HpaB of *E. coli* is an aromatic hydroxylase having a broad substrate specificity range and can hydroxylate 3- HPA, 4HPA, chloro- and methyl-aromatics (e.g., 3-chloro-4HPA, 4-chloro-PA, 4-chlorophenol, 3-chlorophenol, and p-cresol), and dihydroxylated aromatic compounds [e.g., HPC, 2,5-dihydroxyphenylacetic acid, catechol, resorcinol, hydroquinone, and 3,4-dihydroxy phenylalanine (L-DOPA)] [2, 13, 14]. It is remarkable that a single amino acid substitution affected the promiscuous substrate range, which led to the inability to degrade 3HPA and L-tyrosine. Although it is not uncommon that enzyme function and activity can be altered by a single amino acid substitution, to the best of our knowledge, complete loss of degradation activity with respect to native subtrates has been rarely reported, particularly for discriminating between the structural isomers.

Due to its high catalytic efficiency and versatility, HpaB has great potential in biotechnological and pharmaceutical applications [11]. It has been used to produce potential antioxidants of trihydroxyphenolic acids [25]; hydroxylated phenylpropanoids, which have attractive pharmacological properties [14]; and L-DOPA, which is used for the treatment of Parkinson’s disease [26, 27]. Information regarding the substrate specificity that is determined by a single amino acid substitution will contribute to a better understanding of substrate binding and may provide the opportunity to develop biocatalytic hydroxylation processes that require highly-developed substrate specificity.

## Methods

### Strains and culture conditions

*E. coli* REL606 was obtained from Richard E. Lenski, Michigan State University [28], and *E. coli* BL21(DE3) was provided by F. William Studier, Brookhaven National Laboratory [29]. Cells were cultured aerobically in 125-mL flasks containing 25 mL of defined medium at pH 7.0 and incubated at 37 °C with shaking at 200 rpm. M9 medium (6.78 g/L Na_2_HPO_4_, 3 g/L KH_2_PO_4_, 1 g/L NH_4_CL, and 0.5 g/L NaCl) supplemented with 0.8 g/L MgSO_4_·7H_2_O and 0.5 ml/L trace metal solution was used. The trace metal solution contained 2.2 g/L ZnSO_4_·7H_2_O, 1 g/L CuSO_4_·5H_2_O, 0.5 g/L MnSO_4_·4H_2_O, 0.02 g/L Na_2_B_4_O_7_·10H_2_O, 2 g/L CaCl_2_, 0.1 g/L (NH_4_)_6_MO_7_O_24_·4H_2_O, 0.5 M HCl, and 10 g/L FeSO_4_·7H_2_O [30]. For bacterial growth on 3HPA or 4HPA, 3 g/L (20 mM) or 0.76 g/L (5 mM) of 3HPA or 4HPA was added to the medium as the sole carbon source. To evaluate tyrosine utilization, 0.54 g/L (3 mM) L-tyrosine was added to the defined medium supplemented with 3 g/L glucose. Bacterial growth was monitored by measuring absorbance at 600 nm.

### Construction of HpaB-expression vectors

Plasmids and primers used in this study are listed in Table 1 and Table S1 of Additional file 1, respectively. *hpaB* was PCR-amplified from the genomic DNA of *E. coli* BL21(DE3) or REL606 using *hpaB*-F/*hpaB*-R primers. Plasmid pHCE-IIB was used for the constitutive expression of *hpaB*. The pHCE-IIB has a strong constitutive promoter cloned from the thermostable D-amino acid aminotransferase (D-AAT) gene of *Geobacillus toebii* [31]. The ribosomal binding site of the D-AAT promoter was modified to match perfectly with the 3′ end of the *E. coli* 16S rRNA [31]. The purified PCR product and pHCE-IIB vector were digested with both *BamH*I and *Xba*I and were then ligated into the pHCE-IIB vector using T4 ligase. The constructed vectors were electroporated into *E. coli* REL606.

**Table 1:** *E. coli* strains and plasmids used in this study

Strain or plasmid	Description	Source
Strains		
*E. coli* REL606	*E. coli* str. B F^−^, *tsx*-467(Am), *araA230*, *lon*^−^, *rpsL227*(strR), *hsdR*^−^, [*mal*^+^](*LamS*)	Richard E. Lenski
*E. coli* BL21(DE3)	*E. coli* str. B F^−^*ompT gal dcm lon hsdS*_B_(*r*_B_^−^*m*_B_^−^) λ(DE3 [*lacI lacUV5*-*T7p07 ind1 sam7 nin5*]) [*malB*^+^]_K-12_(λ^S^)	F. William Studier
Plasmids		
pHCE-IIB	Expression vector carrying the ampicillin resistance gene and a high-level constitutive expression promoter (HCE)	[31]
pHCE-IIB-HpaB(R379)	pHCE-IIB carrying *hpaB* with arginine at the 379th position [equivalent to *hpaB* of *E. coli* BL21(DE3)]	This study
pHCE-IIB-HpaB(C379)	pHCE-IIB carrying *hpaB* with cysteine at the 379th position (equivalent to *hpaB* of *E. coli* REL606)	This study
pHCE-IIB-HpaB(G379)	pHCE-IIB carrying *hpaB* with glycine at the 379th position (R379G substitution)	This study
pHCE-IIB-HpaB(S379)	pHCE-IIB carrying *hpaB* with serine at the 379th position (R379S substitution)	This study

### Site-directed mutagenesis

*hpaB* of BL21(DE3) [pHCE-IIB-HpaB(R379)] cloned into pHCE-IIB was used as the template for site-directed mutagenesis. Appropriate point mutation (R379S or R379G) was introduced into *hpaB* of *E. coli* BL21(DE3) using the QuikChange™ site-directed mutagenesis kit (Agilent, Santa Clara, CA, USA) in accordance to the manufacturer’s instruction. All the mutated genes were sequenced to check for the desired mutation. The plasmid carrying the appropriate mutation was electroporated into *E. coli* REL606: 1 μl of the constructed plasmid was added to 100 μl of electrocompetent cells in the electroporation cuvette on ice. Electroporation was performed using MicroPulser Electroporator (Bio-Rad Laboratories, Hercules, CA, USA) with a pulse setting of the bacteria mode. Transformants resuspended in 1 ml of LB medium were incubated at 37 °C for 1 h, which were selected on LB agar plates supplemented with ampicillin (100 μg/ml).

### SDS-page

Whole cell lysates of *E.coli* were electrophoresed on SDS-PAGE. Cells were harvested by centrifugation at 13,000 rpm for 3 min. The cell pellet was resuspended in 50 μl of sample buffer [60 mM Tris/HCl (pH 6.8), 25% glycerol, 2% SDS, 0.1% bromophenol blue, and 5% mercaptoethanol] and heated for 10 min at 95 °C to prepare whole cell lysates. The samples were separated by running them on 15% SDS-PAGE gel. The gel was stained with Coomassie blue.

### Quantification of 3HPA and HPC

To quantify 3HPA and HPC in the culture supernatants, LC-TripleQ-MS analysis was performed on Nexera2 LC system (Shimadzu Corp., Kyoto, Japan) combined with a triple quadrupole MS equipped with an electrospray source (LC-MS 8040, Shimadzu Corp., Kyoto, Japan). Briefly, the supernatants of cell cultures were collected and filtered through 0.2-μm polytetrafluoroethylene (PTFE) filters. Samples (5 μL) were injected into a Kinetex C18 column (100 × 2.1 mm, 2.6 μm; Phenomenex, Torrance, CA, USA) at a flow rate of 300 μL/min with a mobile phase containing 0.1% formic acid (solvent A) and acetonitrile containing 0.1% formic acid (solvent B). MS was operated under the following conditions: capillary voltage, − 3000 V; capillary temperature, 350 °C; vaporizer temperature, 300 °C; sheath gas, 3 L/min; ion sweep gas, 2.0 Arb; Aux gas, 10 Arb; and drying gas, 8 L/min. Multiple reaction monitoring (MRM) transitions used for detection of 3HPA and HPC were as follows: 3HPA, 151 > 79.1 (−) and HPC, 167 > 123.05 (−).

### Structural modeling and molecular docking

To locate the geometric 379th postion in HpaB, the three-demensional structure of *E. coli* HpaB (PDB ID: 6 EB0) [15] was visualized using UCSF Chimera [32]. The active site where 4HPA binds to was identified using the crystal structure of the HpaB–FAD–4HPA complex (PDB ID: 2YYJ) [24]. The homology model structure of HpaB from *E. coli* REL606 was generated based on the crystal structure of HpaB of *E. coli* [15] using Modeller v9.21 [33]. The model structure with the lowest DOPE score was selected. Molecular docking of 3HPA and 4HPA into the HpaB structure was performed with AutoDock Vina [34] using the active site of the HpaB–FAD–4HPA complex from *T. thermophilus* HB8 [24]. The docking parameters of exhaustiveness and number of modes were set to 1000 and 100, respectively.

## Supplementary information

**Additional file 1 Table S1.** Primers used for PCR amplification and site-directed mutagenesis of *hpaB*. **FigureS1.** Chemical structures of 3- and 4-hydroxyphenylacetate (HPA). **Figure S2.** Comparison of gene clusters for HPA catabolism in laboratory strains of *E. coli* BL21(DE3), REL606, and W. **Figure S3.** Confirmation of the expression of HpaB variant proteins cloned in pHCE-IIB. **Figure S4.** Growth curves of REL606 expressing *hpaB* variant proteins in the defined medium supplemented with L-tyrosine. **Figure S5.** Molecular docking of HPAs into the HpaB component from the crystal structure of the HpaB–FAD–4HPA complex from *T. thermophilus* HB8.

## Data Availability

All data generated or analyzed during this study are included in this published article and its supplementary information files.

## Abbreviations

HPA: Hydroxyphenylacetic acid
HPC: Homoprotocatechuate
