# Supplementary material for: A single amino acid substitution in aromatic hydroxylase (HpaB) of Escherichia coli alters substrate specificity of the structural isomers of hydroxyphenylacetate
Source: BMC Microbiol. 2020 May 6;20:109. doi: 10.1186/s12866-020-01798-4 (PMC7201708; doi:10.1186/s12866-020-01798-4)

**Table S1.** Primers used for PCR amplification and site-directed mutagenesis of *hpaB*.

| Primer | Oligonucleotide sequence (5′→3′) | Purpose |
| --- | --- | --- |
| *hpaB*-F | CCCCGGATCCAAAGCGACCTCGATCACA | PCR amplification of *hpaB* |
| *hpaB*-R | CGTCTAGATTATTTCAGCAGCTTATCCAGCA |  |
| HpaB(S379)-F | GCCAGTACGCTATAGGTTTGCAGTGCGGCA | Substitution of arginine in the 379^th^ position in *hpaB* of BL21(DE3) with serine |
| HpaB(S379)-R | TGCCGCACTGCAAACCTATAGCGTACTGGC |  |
| HpaB(G379)-F | GCCAGTACGCCATAGGTTTGCAGTGCGGCA | Substitution of arginine in the 379^th^ position in *hpaB* of BL21(DE3) with glycine |
| HpaB(G379)-R | TGCCGCACTGCAAACCTATGGCGTACTGGC |  |

**Figure S1. Chemical structures of 3- and 4-hydroxyphenylacetate (HPA)**


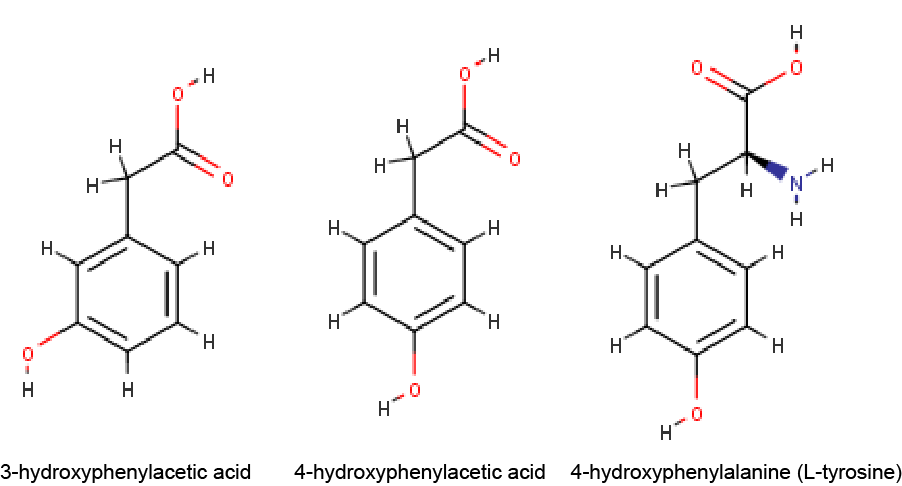


**Figure S2. Comparison of gene clusters for HPA catabolism in laboratory strains of *E. coli* BL21(DE3), REL606, and W.** Genes flanking the *hpa* cluster (*tsr* and *yjiY*) are contiguous in the K-12 genome, and their homologs are colored blue. The *hpa* cluster (colored red) consists of *hpa*_u_ (upper route) and *hpa*_m_ (meta-cleavage route) regions.


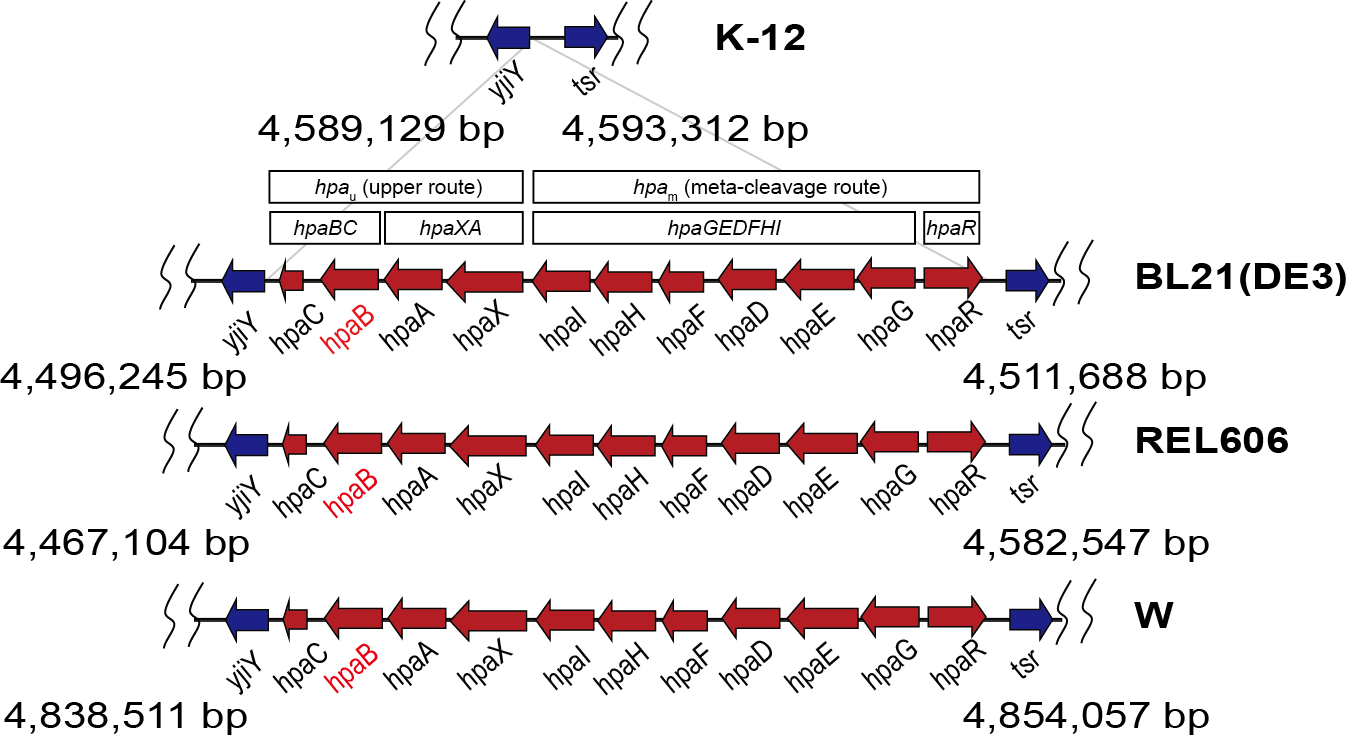


**Figure S3. Confirmation of the expression of HpaB variant proteins cloned in pHCE-IIB.** SDS-PAGE showing expression of HpaB with the different residue at position 379 according to culture times. *E. coli* REL606 transformed with the empty pHCE-IIB serves as a reference (CV). All HpaB variants were expressed in *E. coli* REL606 grown in LB media at 37 °C. Lane M: molecular mass marker.


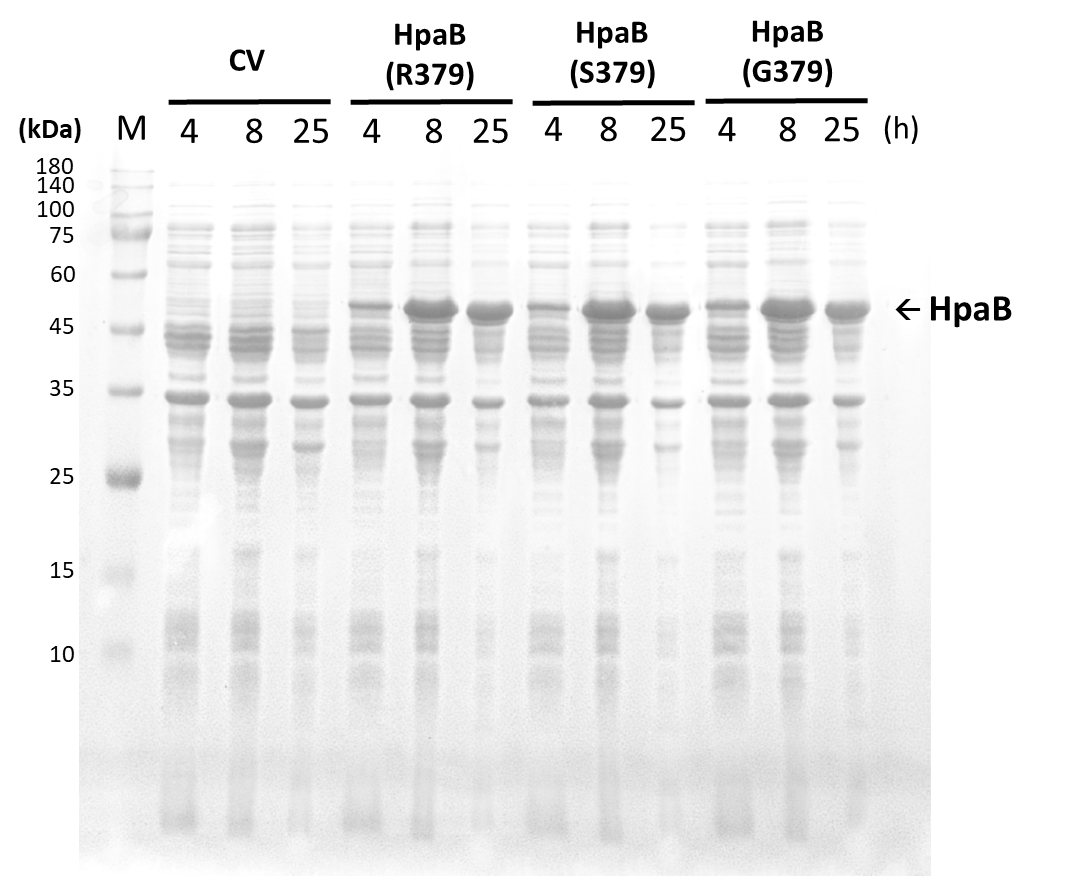


**Figure S4. Growth curves of REL606 expressing HpaB variant proteins in the defined medium supplemented with L-tyrosine.** (**a**) Growth curves. (**b**) Color changes of the culture supernatants after 50 h of incubation. Strains are *E. coli* REL606 cells with pHCE-IIB containing HpaB with the different residue at position 379: C379 (●), G379 (▽), S379 (△), R379 (■). REL606 transformed with the empty pHCE-IIB (⭘) serves as a reference (CV). The culture medium was the defined medium supplemented with 3 g/L glucose and 0.54 g/L (3 mM) L-tyrosine. Only REL606 harboring pHCE-IIB-HpaB(R379) displayed brown coloration in the medium. Error bars denote the standard deviation of mean from three independent cultures.

**A**

**B**


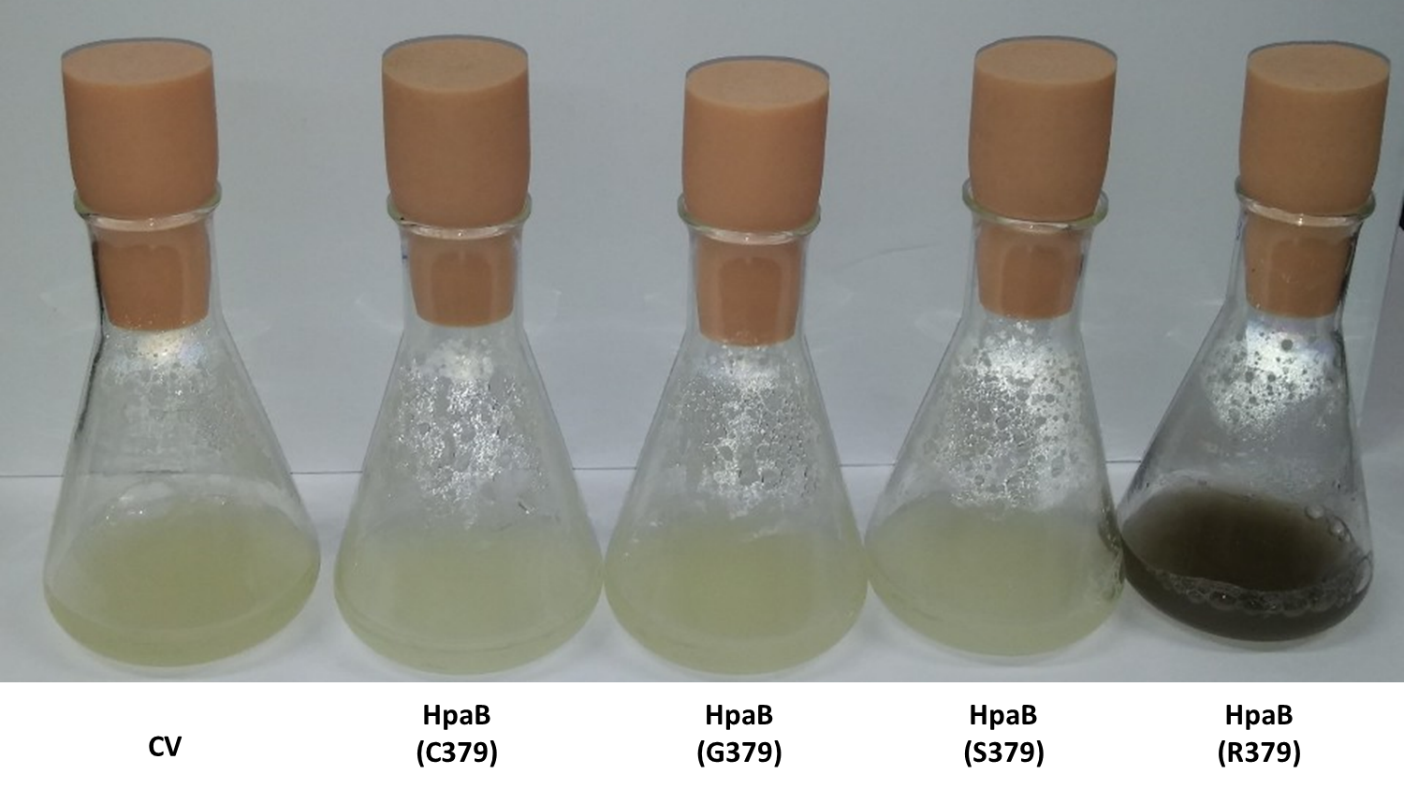


**Figure S5. Molecular docking of HPAs into the HpaB component from the crystal structure of the HpaB–FAD–4HPA complex from *T. thermophilus* HB8.** (**a**) 4HPA binding. (**b**) 3HPA binding. Geometric position of 4HPA from the crystal structure (colored green) was superimposed with the simulated position of 4HPA or 3HPA. Two enlarged images were captured from different angles. The substrate docking simulation shows that the orientation of 3HPA is different from that of 4HPA.


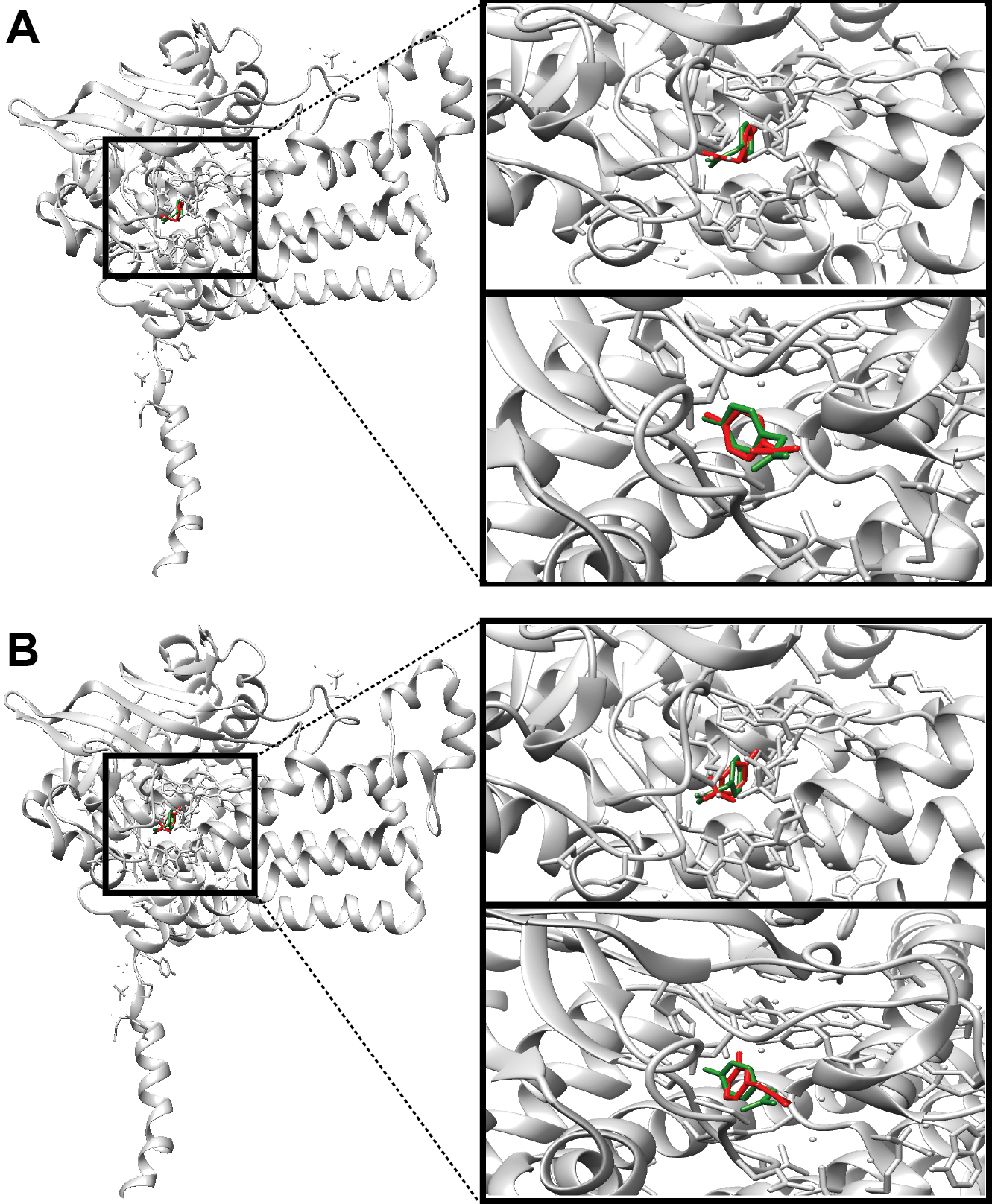

Supplement: Supplementary file 1 — Additional file 1 Table S1. Primers used for PCR amplification and site-directed mutagenesis of hpaB. FigureS1. Chemical structures of 3- and 4-hydroxyphenylacetate (HPA). Figure S2. Comparison of gene clusters for HPA catabolism in laboratory strains of E. coli BL21(DE3), REL606, and W. Figure S3. Confirmation of the expression of HpaB variant proteins cloned in pHCE-IIB. Figure S4. Growth curves of REL606 expressing hpaB variant proteins in the defined medium supplemented with L-tyrosine. Figure S5. Molecular docking of HPAs into the HpaB component from the crystal structure of the HpaB–FAD–4HPA complex from T. thermophilus HB8. [file 12866_2020_1798_MOESM1_ESM.docx]
